# Links Among Crop Diversification, Microbial Diversity, and Soil Organic Carbon: Mini Review and Case Studies

**DOI:** 10.3389/fmicb.2022.854247

**Published:** 2022-04-25

**Authors:** Rachel Wooliver, Stephanie N. Kivlin, Sindhu Jagadamma

**Affiliations:** ^1^Department of Biosystems Engineering and Soil Science, The University of Tennessee, Knoxville, Knoxville, TN, United States; ^2^Department of Ecology and Evolutionary Biology, The University of Tennessee, Knoxville, Knoxville, TN, United States

**Keywords:** above-belowground interactions, agroecosystem, biodiversity-ecosystem function, crop diversification, soil health, soil microbial diversity, soil organic carbon

## Abstract

Interactions between species above- and belowground are among the top factors that govern ecosystem functioning including soil organic carbon (SOC) storage. In agroecosystems, understanding how crop diversification affects soil biodiversity and SOC storage at the local scale remains a key challenge for addressing soil degradation and biodiversity loss that plague these systems. Yet, outcomes of crop diversification for soil microbial diversity and SOC storage, which are key indicators of soil health, are not always positive but rather they are highly idiosyncratic to agroecosystems. Using five case studies, we highlight the importance of selecting ideal crop functional types (as opposed to focusing on plant diversity) when considering diversification options for maximizing SOC accumulation. Some crop functional types and crop diversification approaches are better suited for enhancing SOC at particular sites, though SOC responses to crop diversification can vary annually and with duration of crop cover. We also highlight how SOC responses to crop diversification are more easily interpretable through changes in microbial community composition (as opposed to microbial diversity). We then develop suggestions for future crop diversification experiment standardization including (1) optimizing sampling effort and sequencing depth for soil microbial communities and (2) understanding the mechanisms guiding responses of SOC functional pools with varying stability to crop diversification. We expect that these suggestions will move knowledge forward about biodiversity and ecosystem functioning in agroecosystems, and ultimately be of use to producers for optimizing soil health in their croplands.

## Introduction

Because soil microbes drive soil functioning, their diversity is a major target for improving agricultural soils, which have lost between one-third and one-half of their organic carbon relative to natural vegetation due to conventional management practices (Wei et al., 2014; Tang et al., 2019). Crop diversification is among several management practices used to enhance soil biodiversity and ecosystem functions including soil organic carbon (SOC) storage (Tamburini et al., 2020; Garland et al., 2021). Specifically, greater accumulation of SOC in agroecosystems with greater crop diversity mainly occurs through increases in the quality, quantity, and chemical diversity of plant-derived carbon inputs to soils, thereby fostering the growth and diversity of soil microbial communities which enhance the formation and storage of SOC (Zhang et al., 2021). These patterns align with the positive local-scale relationships observed in natural ecosystems among plant diversity, soil microbial diversity, and SOC (Porazinska et al., 2018; Zhang et al., 2018; Hanif et al., 2019). Still, in practice, responses of soil microbial diversity and SOC to crop diversification are highly variable among agroecosystems (Venter et al., 2016; Tamburini et al., 2020; Beillouin et al., 2021; Hannula et al., 2021). This variation in responses makes management decisions aimed to increase soil health in agroecosystems challenging.

Lessons learned from plant and microbial ecology of natural/unmanaged systems is helpful in explaining site-level differences in relationships among plant diversity, microbial diversity, and ecosystem functioning of managed agroecosystems. In a grassland biodiversity experiment, positive effects of plant diversity on soil fungal diversity occur through increased aboveground biomass production and thus supply of organic substrates (Cline et al., 2018). This means that where plant biomass production is high, fungal richness is also high. However, fungal functional guilds respond differently to plant diversity depending on plant functional type and soil abiotic properties, with direct consequences for the accumulation and persistence of SOC. For example, richness of saprotrophic fungi responds strongly to plant functional diversity (Cline et al., 2018), suggesting that crop diversification efforts that do not effectively increase plant functional diversity will have less effect on saprotroph diversity. Given the functional diversity across saprotrophic fungi (McGuire et al., 2010), higher diversity of saprotrophs in a soil community should increase the community’s ability to break down a greater diversity of organic compounds, thus lowering SOC. Further, richness of arbuscular mycorrhizal fungi (AMF) responds more strongly to plant diversity when legumes are present (Cline et al., 2018; Guzman et al., 2021). Especially in low-quality (low-SOC and low-nitrogen) soils, it may be that the introduction of a legume that provides some available nitrogen increases the ability of AMF to provide more limiting phosphorus resources to plants. More diverse AMF communities provide an additional benefit through enhanced protection of plants from pathogens (Wehner et al., 2011), thereby increasing plant vigor and organic inputs to soils. Though AMF have the ability to decompose SOC (Talbot et al., 2008), they also produce compounds that promote soil aggregation and thus the stabilization of SOC (Wei et al., 2019). However, nitrogen fertilization (a common practice in agroecosystems) may eliminate the usefulness of AMF to plants and can even make them parasitic (Johnson, 1993), therefore decreasing plant biomass production and organic inputs to soils. Ultimately, disproportionate changes in the diversity of any one of these microbial functional groups with crop diversification can potentially change the direction and/or magnitude of change in SOC.

Alongside soil microbe-SOC interactions, experimental factors can influence how ecosystems respond to crop diversification. First, crop species identities are important from a plant functional perspective. Typically, cropping systems with more functionally diverse plants have greater positive effects on SOC relative to monoculture systems, potentially by exploiting niches that would otherwise be unproductive (Fornara and Tilman, 2008; King and Blesh, 2018). However, SOC responses to crop diversification are higher in cases where the focal monoculture crop is not corn (*Zea mays* L.), likely because corn produces higher amounts of biomass with low C:N ratio, which can contribute considerably to SOC accumulation (McDaniel et al., 2014). A meta-analysis revealed that agroforestry on average has a stronger positive effect on SOC compared to cover cropping and crop rotation (19, 13, and 3%, respectively), which is likely linked to the amount of litter inputs (Beillouin et al., 2021). Agroforestry is often not represented in synthesis of crop diversification effects on soil microbial communities and function (Garland et al., 2021), despite known positive influences on soil fauna (Marsden et al., 2020). Second, site-specific responses may cause the same plant diversification regime to vary in ecosystem functioning across field sites, illustrating the importance of local-scale variation in abiotic factors such as soil type and climate on ecosystem response to plant diversity (Spehn et al., 2005). In agroecosystems, tillage and fertilization are two additional abiotic factors that may potentially counteract beneficial effects of crop diversification on soil microbial diversity and SOC responses, though evidence is mixed (McDaniel et al., 2014; Poeplau and Don, 2015; King and Blesh, 2018). Third, slower change in SOC relative to soil microbial diversity introduces a key limitation of study duration on SOC responses to crop diversification, even if microbial diversity has already started to change. Though SOC stock with cover cropping (vs. fallow) increases over time, with an average of 0.32 Mg ha^–1^ per year, many crop diversification studies are limited to less than 10 years during which site-level changes in SOC stock can still be neutral or difficult to detect (Poeplau and Don, 2015). Last, sampling effort can limit the confidence with which we can estimate changes in microbial diversity and SOC. Strategies for estimating soil microbial diversity can be resource-intensive, requiring some tradeoff among (for example) number of samples, sequencing depth, and sequencing length for next-generation sequencing, any one of which can limit coverage of existing soil microbial taxa. Further, because SOC changes very slowly (i.e., in the timespan of years to decades), large sample sizes are necessary to detect changes early on until a sufficiently large change has occurred (Kravchenko and Robertson, 2011).

We discuss how the results of five crop diversification case studies (described in Table 1) fit into current knowledge about linkages among plant diversity, soil microbial diversity, and SOC. We then propose ways forward for understanding and standardizing studies of biodiversity-ecosystem functioning linkage in agroecosystems. We consider both bacterial and fungal communities, along with subsets of fungal taxa whose ecological lifestyles have been assigned (AMF, plant pathogens, and saprotrophs; Nguyen et al., 2016). The select five case studies meet all the following criteria: (1) report changes in SOC, (2) use corn, wheat, (*Triticum aestivum* L.) and/or soybean (*Glycine max* L.) (three globally important row-crops) as a focal crop, (3) use Illumina next-generation sequencing to generate sequence data for bacterial and/or fungal communities in bulk soil, and (4) provide sequencing data publicly or by request. We specifically use studies that provide next-generation sequencing data, following the recent movement toward DNA-based analyses of microbial communities as a metric for soil health (Hermans et al., 2020; Norris et al., 2020; Fierer et al., 2021). We process raw sequence data from each study via the same pipeline to help in standardizing results across studies (Callahan et al., 2016) and analyze resulting taxon abundance tables and raw SOC data to determine how crop diversification alters soil microbial diversity and SOC, along with associations between soil microbial diversity and SOC (Supplementary Methods 1)^1^. Further, we describe context-dependency of experimental factors. Based on these patterns, we make suggestions for the standardization of ongoing work to understand crop diversification effects on soil microbial diversity and SOC.

**TABLE 1:** Case study details.

	Ashworth et al., 2017 *Soil Biol. Biochem.*	Cloutier et al., 2020 *Sci. Rep.*	Gao et al., 2019 *Forests*	Song et al., 2018 *Front. Env. Sci.*	Strom et al., 2020 *Appl. Soil Ecol.*
Study focus	Response of soil bacterial communities to changes in crop sequence and biocovers	Shifts in soil fungal communities across cover cropping treatments, with a focus on AMF	Changes in soil bacterial communities across an agroforestry chronosequence	Shifts in soil microbial communities and soil fertility across cropping systems	How crop rotation shapes soil funga, chemical properties, and soybean cyst nematodes
Location	Tennessee, United States (MTREC: 36°1′ N, 85°7′ W; RECM: 35°32′ N, 88°26′ W)	Pennsylvania, United States (40°43′N, 77°55′W)	Shaanxi, China (34°21′N, 107°43′E)	Heilongjiang, China (49° N, 125°41′E)	Minnesota, United States (44°4′N, 93°33′W)
Soil order	Alfisol	Ultisol	Cambisol	Vertisol	Mollisol
Focal crop	Corn, Soybean, Cotton	Corn, Soybean, Wheat	Wheat	Soybean	Corn, Soybean
Control, **crop diversity treatments**, and *additional treatments* Mono. = monoculture	A. Cotton mono. (RECM only) B. Corn mono C. Soybean mono. D. Corn-soybean rotation X A. Fallow *B. Poultry litter* C. Wheat cover D. Vetch cover	A. Fallow B. Canola cover C. Clover cover D. Oat cover E. Pea cover F. Radish cover G. Rye cover H. Three-species cover I. Six-species cover	A. Wheat mono. B. 5-year-old agroforestry C. 9-year-old agroforestry D. 14-year-old agroforestry	A. Fallow soybean B. Continuous soybean C. Corn-soybean rotation D. Wheat-soybean rotation	A. Corn mono. B. Soybean mono. C. Corn-soybean annual rotations sampled under corn D. Corn-soybean annual rotations sampled under soybean *E. Corn-soybean 5-year rotation* *F. Mono. of other genotypes*
Annual fertilizer rates	128.5 kg N ha^–1^ (corn only) 33.4 kg N ha^–1^ (cotton only) 66.7 kg N ha^–1^ as urea (wheat/fallow cover treatments only) 50.4 kg N ha^–1^ as urea (vetch cover treatment only) 58.7 kg P ha^–1^ as KCl	*(prior to the first corn planting, all plots received 47 Mg ha* ^–^ *^1^ dairy bedded-pack manure; proportion N not stated)*	160 kg N ha^–1^	196.5 kg N ha^–1^ as (NH_4_)_3_PO_4_ and urea 118.2 kg P ha^–1^ as (NH_4_)_3_PO_4_ and K_3_PO_4_ 57.25 kg K ha^–1^ as K_3_PO_4_	224.4 kg ha^–1^ as urea (2015 and 2016 corn only) 89.7 kg ha^–1^ P (2014 only) 134 kg ha^–1^ K (2014 only)
Tillage regime	No-till	Chisel tillage (depth not specified) before cash and cover crop planting	Tillage (type not specified) to 20 cm before cash crop planting	*Not stated*	Chisel tillage (depth not specified) and field cultivation before cash crop planting
Expt. duration	11 and 12 years	3 years	5–14 years	11 years	33 and 34 years
Soil sampling depth	0–15 cm	0–20 cm	0–10 cm	0–20 cm	0–20 cm
Soil sampling season	Spring	Spring, Summer	Fall	Fall	Spring, Summer, Fall
C measurement	SOC (%)	SOM (%) POXC (mg kg^–1^)	SOC (g kg^–1^) DOC (mg kg^–1^) (by treatment)	SOM (g kg^–1^) (by treatment)	SOC (%)
Replicates	3–4	4	3	3	4
Composited subsamples	6	10	*Not stated*	7	*Not stated*
Plot size (m^2^)	75 (MTREC), 57 (RECM)	59	*750*	*Not stated*	*35*

## Current Knowledge and Case Studies

### Variation Across Crop Species Identity and Diversity

Generally, diversification of plant communities leads to higher ecosystem functioning. This pattern is commonly attributed to *niche complementarity effects* (Eisenhauer, 2012), wherein coexisting plant species capitalize on different limiting resources across space or time, thereby maximizing resource use and biomass production (Tilman, 1999). There is a strong microbial component to such effects because plant diversity can change microbial communities in ways that feed back to enhance plant performance (Zak et al., 2003). Positive interactions among plant diversity, microbial diversity, and soil functioning can be especially strong in high-stress ecosystems, including early-successional alpine ecosystems where soils are C-deficient (Porazinska et al., 2018) and managed grasslands (Lange et al., 2015). Thus, it is not surprising that similar patterns have been identified in agroecosystems (Tamburini et al., 2020; Garland et al., 2021). However, multiple factors can prevent positive interactions among plant diversity, microbial diversity, and ecosystem functioning in agroecosystems. First, key microbial functional groups, such as pathogens and saprotrophs, can negatively affect SOC accumulation by reducing carbon inputs to soils from plants (van der Heijden and Wagg, 2013) and mineralizing soil carbon (Heijden et al., 2008) respectively. Second, just as in natural ecosystems (De Deyn et al., 2008), plant functional types (e.g., grass vs. legume) and life histories (i.e., annual vs. perennial) can mediate soil microbial communities and SOC storage in agroecosystems (Zhang et al., 2021), making them important to consider when implementing crop diversification (King and Blesh, 2018). For example, crop rotation increases SOC compared to soybean monocultures (McDaniel et al., 2014; Chatterjee et al., 2016). However, crop rotation does not increase SOC compared to corn monoculture (McDaniel et al., 2014; Chatterjee et al., 2016). This is attributed to higher production of low-quality residue biomass by corn that is slowly decomposed compared to soybean (Smil, 1999). Along the same lines, agroforestry, where a (perennial) tree is added to any cropping system, usually accumulates more SOC compared to other crop diversification strategies that introduce only annual species (Beillouin et al., 2021), likely due to year-round, multi-year addition of root and litter carbon. Still, across a gradient of crop species diversity in European cereal (mostly wheat)-based systems (which produces less residual biomass than corn), there is a 5–10 species threshold for maximizing soil bacterial diversity and multifunctionality (Garland et al., 2021). Below, we describe how crop functional types and microbial functional groups explain our findings from five case studies.

**FIGURE 1:**
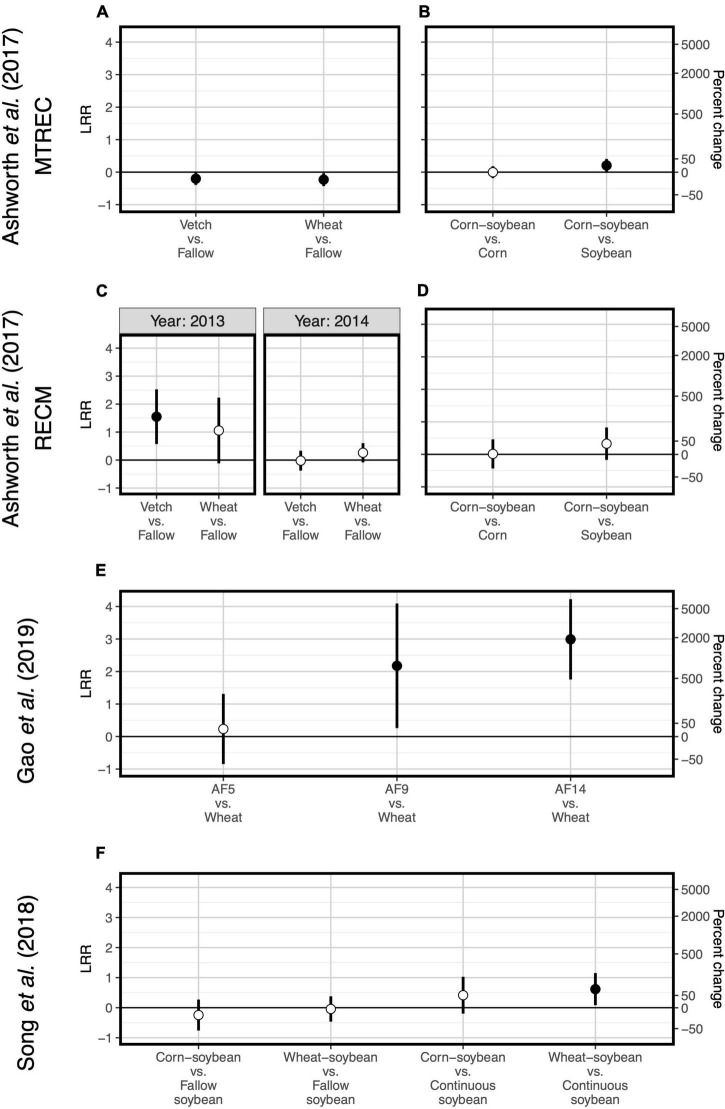
Responses of soil bacterial diversity to crop diversification. Three studies are represented: Ashworth et al. (2017) **(A–D)**, Gao et al. (2019) **(E)**, and Song et al. (2018) **(F)**. Responses are shown as log response ratios (LRR) with 95% confidence intervals which account for low sample sizes (Pustejovsky, 2018). Responses are calculated using Inverse Simpson diversity. Open points represent log response ratios whose 95% confidence intervals overlap zero, while solid points represent log response ratios whose 95% confidence intervals do not overlap zero.

**FIGURE 2:**
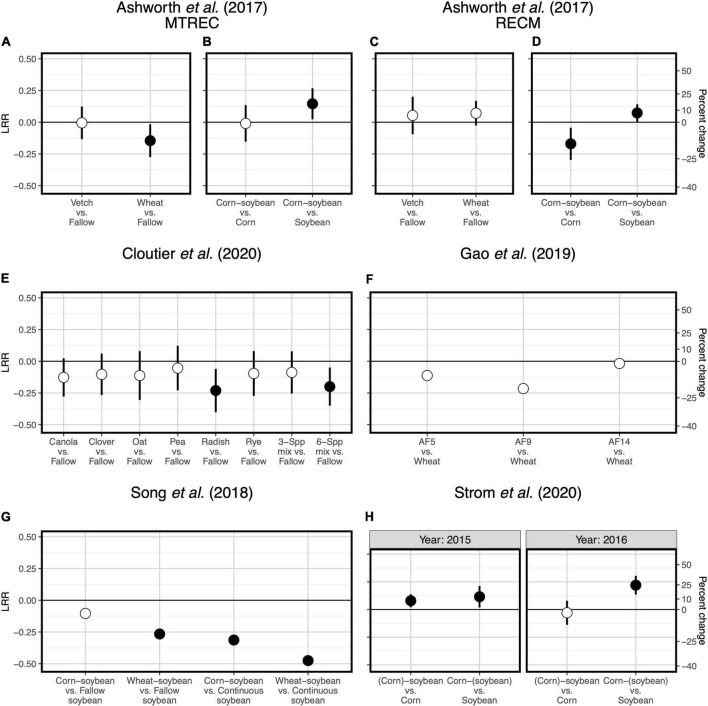
Responses of soil organic carbon to crop diversification. Five studies are represented: Ashworth et al. (2017) **(A–D)**, Cloutier et al. (2020) **(E)**, Gao et al. (2019) **(F)**, Song et al. (2018) **(G)**, and Strom et al. (2020) **(H)**. Responses are shown as log response ratios (LRR) with 95% confidence intervals which account for low sample sizes (Pustejovsky, 2018). Open points represent log response ratios whose 95% confidence intervals overlap zero, while solid points represent log response ratios whose 95% confidence intervals do not overlap zero. Responses in **(F,G)** do not have 95% confidence intervals because the original studies reported only treatment mean organic carbon values rather than plot-level values; solid points represent cases in which the original study found sign.

**FIGURE 3:**
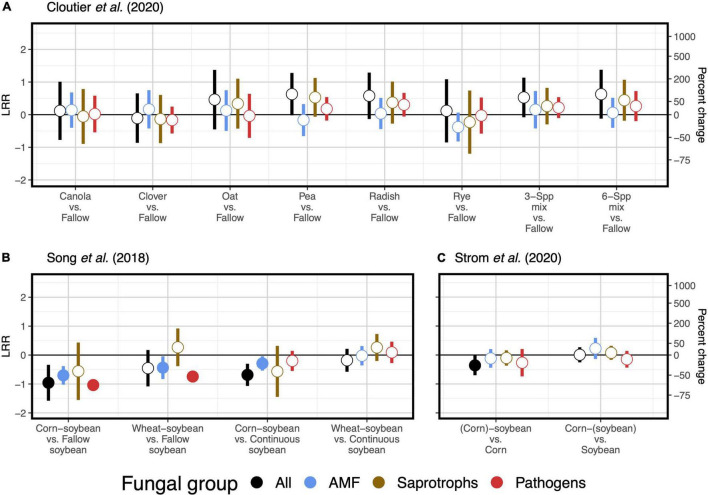
Responses of soil fungal diversity to crop diversification. Responses are shown as log response ratios (LRR) with 95% confidence intervals which account for low sample sizes (Pustejovsky, 2018). Three studies are represented: Cloutier et al. (2020) **(A)**, Song et al. (2018) **(B)**, and Strom et al. (2020) **(C)**. Responses are calculated using Inverse Simpson diversity. While black responses represent the total fungal community, colored responses represent functionally distinct subsets of the fungal community: arbuscular mycorrhizal fungi (AMF; blue), saprotrophic fungi (brown), and plant pathogenic fungi (red). Open points represent log response ratios whose 95% confidence intervals overlap zero, while solid points represent log response ratios whose 95% confidence intervals do not overlap zero.

Two case studies demonstrate that plant functional type can guide microbial diversity and SOC responses to crop diversification. For example, one study implemented a long-term (11- and 12-year) crop diversification experiment in corn and soybean cropping systems in the southeastern United States (Ashworth et al., 2017). Along with other studies supporting selection effects (McDaniel et al., 2014; Chatterjee et al., 2016), introducing corn as a rotational crop to a monoculture soybean system led to more positive responses of bacterial diversity (Figures 1B,D) and SOC (Figures 2B,D) compared to the introduction of soybean as a rotational crop to corn monoculture (Ashworth et al., 2017). A similar pattern occurred for total fungal diversity and SOC in a three-decade-long corn-soybean cropping system in the midwestern United States (Strom et al., 2020; Figures 2H, 3C).

**FIGURE 4:**
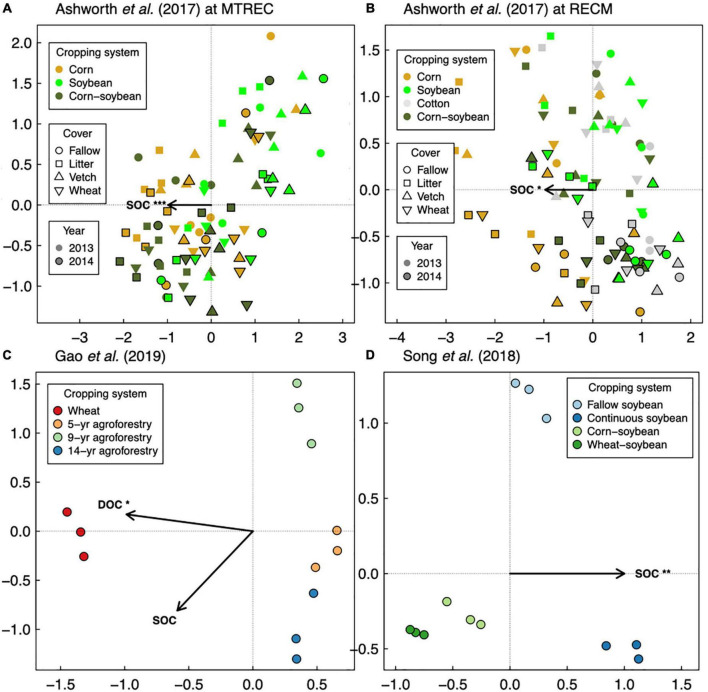
Distance-based redundancy analysis of soil bacterial communities in response to soil organic carbon (SOC), with readily available SOC pools shown if data were available as dissolved organic carbon (DOC). Three studies are represented: Ashworth et al. (2017) **(A,B)**, Gao et al. (2019) **(C)**, and Song et al. (2018) **(D)**. Asterisks represent significance of marginal treatment effects (***p < 0.001, **p < 0.01, *p < 0.05).

Responses of soil microbial diversity and SOC to crop diversification, however, do not always follow similar responses based on plant functional type, but rather those responses are manifested through changes in soil microbial community composition. For example, in a wheat-based system in northwestern China, although effects of walnut (*Juglans regia* L.)-based agroforestry on bacterial diversity are positive overall (increasing diversity up to 17-fold relative to monoculture wheat, Figure 1E), effects on SOC were neutral (Gao et al., 2019; Figure 2F). It is possible that agroforestry does not increase SOC relative to monoculture wheat because soil samples were taken from crop alleys which may receive lower leaf- and root-derived carbon inputs from trees compared to tree alleys (Gao et al., 2019), however this seems unlikely given the clear stimulation of bacterial diversity and alteration of bacterial communities (Figure 4C) by agroforestry treatments. All crop diversification experiments, in fact, drive clear changes in microbial community composition for both bacteria and fungi (Supplementary Table 3), a pattern that is consistent with those identified in grassland plant diversity experiments (Prober et al., 2015). Rather, the authors show that the relative abundance of Actinobacteria (a bacterial group that is known to drive lignocellulose decomposition, Wang et al., 2016) increases with agroforestry, which may in part explain decreases in SOC. In another case study, a 12-year crop diversification experiment in northern China, rotation of soybean with either corn or wheat does not affect bacterial diversity (Figure 1F), decreases fungal diversity (by up to 60%, Figure 3B), and decreases SOC (by up to 40%; Song et al., 2018; Figure 2G) compared with monoculture soybean. One possible explanation for reduced SOC under rotation is that the reduced diversity of AMF led to lower phosphorus mining from soils, thereby increasing available phosphorus with rotation. In the original paper, the authors find that greater soil available phosphorus if correlated with higher relative abundances of Actinobacteria and Ascomycota (Song et al., 2018), which act together to stimulate organic matter decomposition. Overall, these results suggest that crop diversification-driven changes in microbial community composition (Supplementary Table 3) can be more helpful in understanding changes in SOC than microbial diversity alone.

Last, two case studies suggest that for cover crop diversification, functional type of added cover crops is more important than their diversity in driving changes in SOC. In a 3-year-long cover crop diversification experiment within a corn-soybean-wheat rotation system in the northeastern United States (Cloutier et al., 2020), cover crop treatments containing radish (*Raphanus sativus* L.) alone or in a six-species mixture decrease SOC by about 20% relative to a fallow control (Figure 2E). Though none of cover crop treatments affects diversity of all fungi or any of the fungal functional groups (Figure 3A), there are differences in fungal community composition among cover crop treatments (Supplementary Table 3). This suggests that radish may change the abundance of fungal groups that influence organic matter decomposition. Ultimately, these results show that plant function can be more important than plant diversity *per se* in affecting SOC. Ashworth et al. (2017), in addition to manipulating species diversity through crop rotation, adopt different winter cover crop species and found that the effects of cover crops on bacterial diversity and SOC are highly variable across space and time (Figures 1A,C, 2A,C), which we discuss in the following section. Overall, results from these case studies indicate that the 5–10 species threshold for maximizing soil microbial diversity and SOC (Garland et al., 2021) is not a general occurrence, but rather highlight the importance of selecting ideal crops functional types (as opposed to focusing on plant diversity), when considering diversification options.

### Field-Level Responses to Crop Diversification Across Space and Time

Biodiversity-ecosystem functioning experiments show that spatial and temporal variation are important drivers for plant diversity effects on ecosystem functions. Relationships between plant diversity and organic matter decomposition, for example, vary among experimental grassland sites due to differences in soils and indirect effects of plant species on soil conditions (Spehn et al., 2005), and such patterns tend to grow stronger through time (Spehn et al., 2005; Qiu and Cardinale, 2020). However, row-crop systems are much more heavily managed than grasslands, and generally follow an annual cycle where crops are terminated on a seasonal or yearly basis. The combination of management practices and annuality of row-crop systems, therefore, may limit the magnitude of SOC accumulation and changes in microbial communities under crop diversification regimes. Another temporal factor that can be important for the functioning of cropping systems is duration of cover crop throughout the year (Garland et al., 2021). Specifically, after controlling for spatial variation in abiotic factors (climate, soil texture, and soil pH), higher duration of cover crop leads to higher soil bacterial diversity and soil multifunctionality across European agricultural fields, while crop diversification (through crop rotation and/or cover cropping) has no effect on either response variable (Garland et al., 2021). Here, we describe site-to-site variation, seasonal and annual variation, and effects of duration of crop cover for the outcomes of five crop diversification case studies.

One case study allows us to explore site variation in effects of crop diversification. Ashworth et al. (2017) replicate their experiment at each of two sites: University of Tennessee’s Middle Tennessee Research and Education Center (MTREC) in middle Tennessee (36°1′ N, 85°7′ W; annual precipitation = 1,440 mm; mean annual temperature = 14.2°C) and University of Tennessee’s Milan Research and Education Center (RECM) in western Tennessee (35°32′ N, 88°26′ W; annual precipitation = 1,070 mm; mean annual temperature = 14.8°C). Sites are separated by a distance of 185 kilometers. Though responses of bacterial diversity and SOC to crop rotation show similar patterns between sites, responses to cover cropping does not. At MTREC, adoption of either hairy vetch (*Vicia villosa* L.) or wheat (*Triticum aestivum* L.) cover crop in the winter fallow corn-soybean cropping systems significantly decreases soil bacterial diversity (by about 20%; Figure 1A), with wheat cover significantly decreasing SOC by about 14% relative to the fallow treatment (Figure 2A). However, at RECM, adoption of either cover crop tends to increase bacterial diversity (Figure 1C), with neutral effects on SOC (Figure 2C). Notably, the decrease in SOC under wheat cover cropping at MTREC is associated with what the authors found to be a significant increase in the relative abundance of Verrucomicrobia at this site (Ashworth et al., 2017). Despite their ubiquity in soils, members of the phylum Verrucomicrobia are relatively less-studied compared to other bacterial phyla, their free-living oligotrophic (specialized for low-resource environments) lifestyle and association with carbohydrate metabolism genes suggests that they can degrade more recalcitrant carbon compounds (Fierer et al., 2013). Altogether, these results demonstrate the dependencies of cover cropping effects on SOC as determined by site and cover crop identity.

Overall, our case studies suggest that while microbial diversity and SOC responses do not vary at the temporal scale of season, they can vary annually and depend on duration of crop cover. If duration of crop cover is more important than the cover crop itself as previously suggested (Garland et al., 2021), we should expect more positive effects of crop diversification when comparing to a fallow control vs. comparing to a “continuous” control in which the focal crop is grown throughout the fallow season. Such a pattern is indeed apparent for SOC in a soybean-based field experiment (though responses were negative overall, Figure 2G), but not for bacterial or fungal diversity (Song et al., 2018; Figures 1F, 3B). Case studies that measure soil responses at multiple timepoints during the year (Cloutier et al., 2020; Strom et al., 2020) do not show that season plays an important role in soil microbial diversity and SOC responses to crop diversification (Supplementary Table 2). However, rotating soybean with corn significantly increases SOC relative to corn monocultures in the first year of sampling (2015) but has a neutral effect in the following year (2016, Strom et al., 2020; Figure 2H). Further, in the Ashworth et al. (2017) dataset, we see an effect of sampling year at RECM on the responses of bacterial diversity to cover cropping: responses are on average more positive in 2013 (especially under hairy vetch, which increases bacterial diversity by up to threefold) compared to 2014 (Figure 1C). The authors offer that the weather prior to sampling was wetter and warmer and soil microbial communities were more diverse at RECM in 2013 compared to 2014 (Ashworth et al., 2017), suggesting that cover crops are more beneficial for cultivating bacterial diversity under less stressful conditions. Notably, results from one case study suggest that effects of crop diversification on soil microbial diversity and SOC can become more positive across multi-year intervals. In the agroforestry dataset of Gao et al. (2019), walnut-wheat systems have increasingly positive effects on soil bacterial diversity after 9 and 14 years (increasing diversity by about 8- and 19-fold, respectively) relative to wheat monocultures (Figure 2E). Though SOC does not respond significantly to agroforestry integrated system at any point during this chronosequence (Figure 2F), dissolved organic carbon decreases significantly at all timepoints (by 30–40%) relative to wheat monocultures (Figure 4C), indicating that readily available C pools are more sensitive to the inclusion of agroforestry practices than total SOC. While the shortest-duration (3-year) case study shows overall negative effects of cover cropping on SOC (Cloutier et al., 2020), the case study with the longest experimental duration (over three decades, Strom et al., 2020) shows significant increases in SOC in three out of four comparisons between crop rotation and monoculture (8–25% increase, Figure 2H). Combined, these results suggest that both time and space can have critical effects on soil responses to crop diversification.

### Microbial Biodiversity-Ecosystem Function Relationships

**FIGURE 5:**
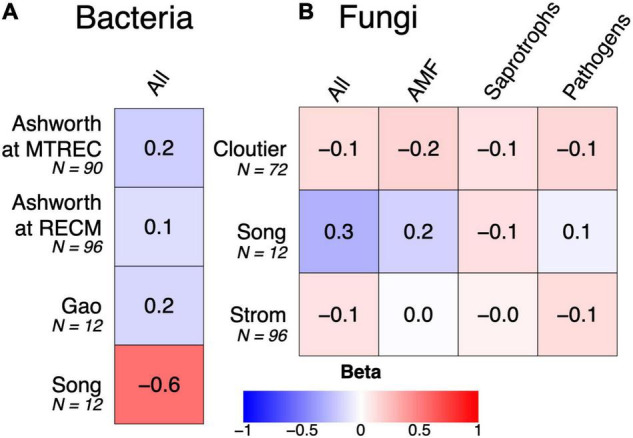
Associations between soil organic carbon and either soil bacterial **(A)** or fungal diversity **(B)**. Associations are shown as standardized beta values derived from linear mixed models that account for study design (i.e., include replicate as a random effect). Colors indicate the magnitude and direction of beta value, from strongly negative (red) to strongly positive (blue). No beta values were significant at α = 0.05.

**FIGURE 6:**
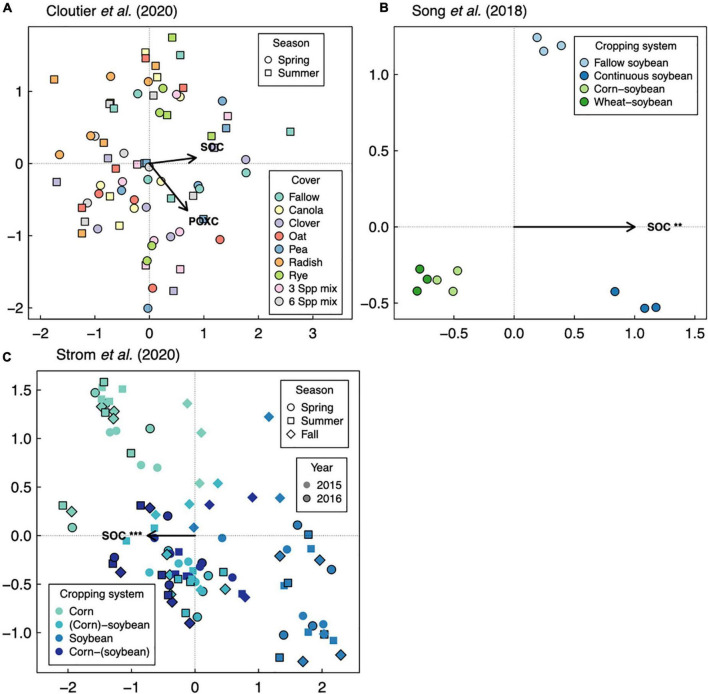
Distance-based redundancy analysis of soil fungal communities in response to soil organic carbon (SOC), with readily available SOC pools shown if data were available as permanganate-oxidizable carbon (POXC). Three studies are represented: Cloutier et al. (2020) **(A)**, Song et al. (2018) **(B)**, and Strom et al. (2020) **(C)**. Asterisks represent significance of marginal treatment effects (***p < 0.001, **p < 0.01).

Soil microbial diversity is emerging as a suggested indicator of soil health due to microbes’ sensitivity to management practices and controls over soil function (Stott, 2019). However, relationships between diversity and ecosystem functioning can be limited in microbial communities due to functional redundancy or stronger effects of community composition on ecosystem functioning (Nielsen et al., 2011). Consistent with this pattern, we implemented linear mixed models testing for associations between SOC and bacterial or fungal diversity (described in Supplementary Methods 1) from our sequence analysis (summarized in Supplementary Table 1) and found no strong positive relationships within the five case studies: rather, the strongest relationship was negative (Figure 5). Instead, all case studies show strong associations between SOC and microbial community composition (Figures 4, 6). There are multiple cases (discussed in the previous subsections) where responses of SOC to crop diversification treatments coincide with changes in the relative abundances of soil microbial groups that are known to directly mediate SOC cycling. For example, reduced SOC under rotation compared to monoculture soybean coincides with increased relative abundances of Actinobacteria and Ascomycota (Song et al., 2018). Altogether, this suggests that the *composition* of soil microbial communities is more helpful than their *diversity* for understanding how management practices such as crop diversification influences SOC.

## Suggestions for Standardization of Future Crop Diversity Experiments

### Optimize Sampling Effort and Sequencing Depth

Ideally, crop diversification would drive effects on soils that are of high magnitude and thus easily detectable with minimal sampling effort. However, soils are highly variable across time and space, which may obscure true effects of crop diversification when sampling effort is insufficient. For example, in surface (0–20 cm) soils of a long-term tillage experiment (Syswerda et al., 2011), within-plot variance in SOC contributes 80% of total variance in SOC across all of six 1-hectare (10,000 m^2^) plots. One way to optimize sampling effort is to increase subsamples collected and homogenized for each plot. In the Syswerda et al. (2011) example, at least 14 subsamples would need to be taken within each plot in order to detect a 10% change in SOC between chisel-plowed and no-till treatments (Kravchenko and Robertson, 2011). The case studies that we have discussed have much smaller plot sizes, as low as 35 m^2^, and thus the numbers of composited subsamples for SOC (Table 1) should be sufficient if spatial variation reflects that of Syswerda et al. (2011). However, subsurface soils are much more variable (e.g., 20–100 cm, Kravchenko and Robertson, 2011) and could be important for storing SOC with longer residence time (Cotrufo et al., 2013). Vertical variation in SOC responses to crop diversification, then, represents a major area of needed future work (see our discussion in the following section).

Even if sampling effort within plots is sufficient, lack of field replication (i.e., number of blocks) can prevent detection of significant changes in SOC and microbial community diversity. Soil microbial diversity, in particular, can vary significantly at local or field-scales (Fierer and Jackson, 2006). However, studies of soil microbial communities in agroecosystems generally use treatment replication sizes of no more than three or four (McPherson et al., 2018; Hannula et al., 2021; Table 1). Given that crop diversification enhances variation in litter quality and chemical complexity, and thus niche space for soil microbes, we should expect simultaneous increases in microbial diversity (Torsvik and Øvreås, 2002). In actuality, current treatment replication does not give sufficient power to detect significant increases in microbial diversity for at least one of our case studies (Box 1). That is, Gao et al. (2019) used three field replicates to determine effects of crop diversity in agroforestry system on soil bacterial diversity, and our reanalysis showed that these effects increased in magnitude over time but were only significant after at least 9 years of agroforestry (**Figure 1K**). We performed an additional power analysis to identify how many replicates it would take to detect a significant effect after only 5 years of agroforestry given the observed effect size, and found that replication would have to increase by two orders of magnitude (Box 1). Thus, we may need to invest much more in sampling effort to detect short-term subtle effects of crop diversification on soil microbial diversity and functions. The example of Gao et al. (2019) also illustrates the issue of temporal variation in soil responses to crop diversification. Moving forward, as next-generation sequencing costs (currently $30/sample) continue to decrease, higher replication will provide higher power in detecting soil microbial diversity responses in early years of experimental implementation. Otherwise, we should leverage long-term field experiments whose effects may be much stronger. Building on this, future work should perform multi-year analysis because responses of soil microbial diversity and SOC can vary annually (Figures 1C, 2H).

**Box 1**

Sample size for soil microbial characterization. Sample size (i.e., replication) is one factor to consider to robustly measure diversity of soil microbial communities. To determine how soil sample size influences the ability to detect crop diversification effects on soil microbial diversity, we conducted a power analysis of results from Gao et al. (2019) using WebPower version 0.6 (Zhang and Yuan, 2018) in R version 4.0.3 (R Core Team., 2020). Cohen’s D was calculated for soil bacterial inverse Simpson diversity in response to 5, 9, and 14 years of agroforestry compared to wheat monocultures as (mean_diversified_–mean_simplified_)/pooled standard deviation. Using these observed effect sizes and an alpha level of 0.05, we determined the achievable statistical power given a range of sample sizes from 2 to 100 + (Box Figure 1). This analysis showed that sample sizes would need to be 103 to achieve sufficient statistical power (0.8; Crawley, 2012) to detect the observed 30% increase in soil bacterial diversity in response to 5-year agroforestry. For 9- and 14- year agroforestry, required sample sizes were lower (16 and 5, respectively) due to higher observed increases in soil bacterial diversity (553 and 1,853%, respectively).

**BOX FIGURE 1:**
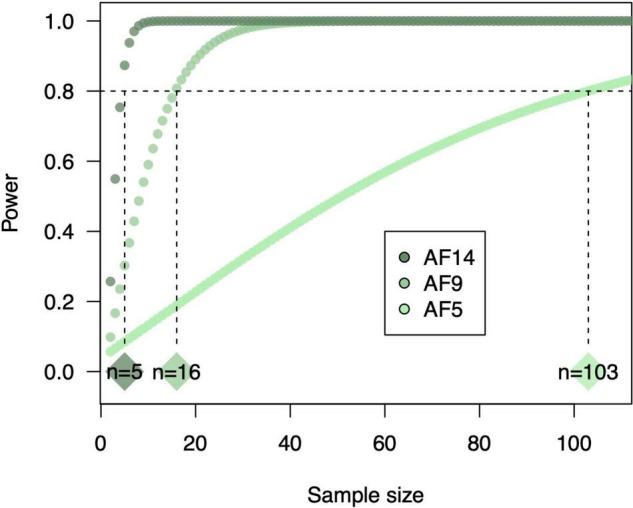
Power curves for responses of soil bacterial diversity to a chronosequence of agroforestry compared to wheat monocultures. The chronosequence included 5, 9, and 14 years of agroforestry (AF5, AF9, and AF14, respectively).

Additionally, when characterizing soil microbial communities, it is important to consider optimizing sequencing depth (i.e., read length and number of reads) and accounting for relic DNA. Inadequate sequencing depth would lead to underestimation of actual diversity, whereas excess sequencing depth would be a resource intensive effort. The case studies highlighted here obtained paired end sequence read lengths of 250–300 bases per sequence, which should be sufficient for 16S gene regions and for most ITS gene regions (Klindworth et al., 2013; Taylor et al., 2016). However, case studies vary by an order of magnitude in average per-sample reads after sequence processing, from under 21,000 (Gao et al., 2019; Cloutier et al., 2020) to more than 150,000 (Ashworth et al., 2017; Song et al., 2018). Species abundance curves are one way to determine whether sequencing depth (number of reads) is sufficient to represent all present microbial taxa (Box 2). Thus, given such apparent variation in soil microbial diversity, we suggest that future work should perform pilot tests of overall diversity to determine proper sequencing depth. Still, the DNA that we aim to sequence for living microbes in the soil may be contaminated with DNA from dead microbes (relic DNA), which can represent up to 50% of total bacterial DNA in soils (Lennon et al., 2018). There is mixed evidence as to whether relic DNA obscures true differences in composition and diversity of soil microbes (Lennon et al., 2018; Carini et al., 2020), and further work should address this issue in agroecosystems.

**Box 2**

Sequencing depth for soil microbial characterization. To illustrate the importance of sequencing depth in understanding soil microbial diversity, we plotted species abundance curves of fungal amplicon sequence variants (ASVs) identified in Cloutier et al. (2020) and Strom et al. (2020) datasets (Box Figures 2A,B respectively). Leveling off of curves in **(A)** indicates sufficient sampling depth. The absence of leveling off in **(B)** indicates insufficient sampling depth, which may hinder insights gained from this dataset about how soil microbial diversity responds to crop diversification. Given this variation in apparent soil microbial diversity in agroecosystems, future studies would benefit from pilot tests to determine the adequate sequencing depth for overall diversity in the systems.

**BOX FIGURE 2:**
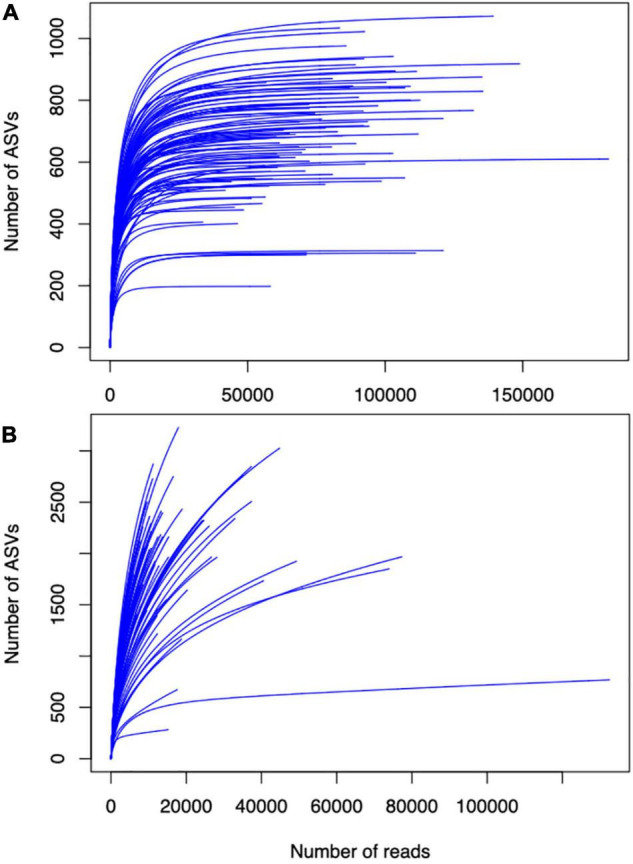
**(A)** Species abundance curves of fungal amplicon sequence variants identified in Strom et al. (2020) and **(B)** Cloutier et al. (2020) datasets.

### Mechanisms of Soil Organic Carbon Accumulation

Identifying coordinated changes (or lack thereof) in SOC and soil microbial diversity can advance our understanding of diversity-ecosystem functioning relationships in agroecosystems, but determining the mechanisms that underlie SOC changes can help us to explain such relationships. Carbon cycling frameworks have increasingly understood microbial influences on SOC storage, but reconciling this framework with changes to SOC under crop diversification remains a major challenge. That is, we have seen a common pattern where corn production systems accumulate more SOC than soybean systems. However, according to the Microbial Efficiency-Matrix Stabilization (MEMS) framework, higher-quality (high percent-nitrogen, low carbon-to-nitrogen ratio) litter (such as that of soybean) decomposes more quickly, thereby increasing readily available carbon (e.g., dissolved organic carbon), which can then be stabilized and protected through associations with the mineral soil matrix (Cotrufo et al., 2013). This mineral-protected SOC, has low turnover (decades to centuries) compared to readily available SOC (weeks to years) and thus contributes to long-term carbon sequestration (Srivastava et al., 2016). Alternatively, lower-quality litter (such as that of corn) decomposes less quickly, resulting in a greater proportion of carbon lost through microbial respiration and higher amounts of readily available carbon in the form of undecomposed plant material (particulate organic carbon, Cotrufo et al., 2013; Lavallee et al., 2020). The case studies that we have discussed here mostly measured only total SOC (except two cases which also measured readily available carbon in the form of dissolved organic carbon or permanganate-oxidizable carbon), so we may have overlooked any changes in SOC that could be representative of mineral-associated vs. particulate SOC. Also, available meta-analysis of crop diversification effects on SOC report changes in only total SOC or readily available SOC in the form of microbial biomass carbon (McDaniel et al., 2014; Poeplau and Don, 2015; Jian et al., 2020) and we suggest future crop diversification studies to quantify not only total SOC but its distribution and stabilization in pools with varying stability, such as particulate SOC and mineral-associated SOC, to address whether the MEMS framework applies in agricultural systems.

Two case studies presented here measure readily available fractions of SOC (Gao et al., 2019; Cloutier et al., 2020), which can be precursors to mineral-protected SOC, according to the MEMS framework. Readily available SOC also shows more sensitivity to soil health and crop yield than total SOC (Lal, 2006; Stott, 2019). The Cloutier et al. (2020) dataset shows that permanganate-oxidizable carbon decreases by about 15% with addition of radish and a six-species mixture (which included radish) as cover crops, a pattern that mirrors the responses of total SOC (shown in Figure 2E). This suggests that radish could ultimately reduce mineral-associated SOC, but evidence for this is mixed. A separate experiment identified increases in permanganate-oxidizable carbon (but no effect on total SOC) with addition of radish as a cover crop in a corn silage system in the eastern United States, which was attributed to low C:N ratio and fast decomposition of radish biomass (Wang et al., 2017). Gao et al. (2019) dataset shows that agroforestry significantly decreases dissolved organic carbon (by 30–40%) relative to monoculture wheat, a pattern which contrasts the neutral responses of total SOC (Figure 2F). Combined with significant increases in soil bacterial diversity after 9 years, these results suggest that agroforestry can cultivate a higher taxonomic diversity of bacteria that immobilizes available carbon into microbial biomass, however this has not yet affected total SOC storage. While readily available SOC changes across treatments, neither study looked at the changes in mineral-associated SOC, which is influenced by the readily available SOC inputs. This points to the need for future work to elucidate SOC dynamics with crop diversification by measuring SOC functional pools with varying stability.

## Conclusion

Recent global analysis has identified strong positive linkages among plant diversity, soil microbial diversity, and SOC in agroecosystems, but these linkages at the site level are quite idiosyncratic. Here, we have discussed contingencies upon plant functional type, number of crop species, field location, and experimental duration in determining how soils respond to crop diversification. Overall, we have described the role of crop functional type (more so than number of species) in how soils respond to crop diversification, with corn contributing more to SOC accumulation in agroecosystems compared to soybean and wheat. Still, higher microbial diversity is not always an outcome of crop diversification or an indicator of increased SOC, and site-to-site and year-to-year variation can introduce significant inconsistency in the effectiveness for any one crop diversification strategy. Though crop diversification does not have consistent effects on soil microbial diversity, it does consistently shift microbial community composition. Further, soil microbial diversity has no strong associations with SOC within sites, but SOC is generally a significant predictor of soil microbial community composition. Thus, despite the limited scope of our analysis of five case studies, we find strong evidence that microbial diversity is a less important mediator of crop diversification effects on SOC compared to microbial community composition. Moving forward, given a combination of more subtle effects that occur in early years of implementation of crop diversification and inherent spatial and temporal variation in SOC and soil microbial communities, we need to optimize sampling efforts and sequencing depths that aid robust estimates of change. We also need to integrate agroecosystems into current theory on soil microbe-carbon interactions. Such efforts will improve our understanding of plant diversity-ecosystem functioning in agroecosystems.

## Author Contributions

RW, SK, and SJ conceived the project. RW acquired and processed the raw sequence data with substantial input from SK. RW conducted statistical analyses. All authors contributed to writing of the manuscript.

## Conflict of Interest

The authors declare that the research was conducted in the absence of any commercial or financial relationships that could be construed as a potential conflict of interest.

## Publisher’s Note

All claims expressed in this article are solely those of the authors and do not necessarily represent those of their affiliated organizations, or those of the publisher, the editors and the reviewers. Any product that may be evaluated in this article, or claim that may be made by its manufacturer, is not guaranteed or endorsed by the publisher.

## Footnotes

1
https://github.com/rwoolive/microbe-cropdiv
