# Supplementary material for: Links Among Crop Diversification, Microbial Diversity, and Soil Organic Carbon: Mini Review and Case Studies
Source: Front Microbiol. 2022 Apr 25;13:854247. doi: 10.3389/fmicb.2022.854247 (PMC9082997; doi:10.3389/fmicb.2022.854247)
Supplement: Supplementary file 1 [file Data_Sheet_1.docx]

***Supplementary Material***

Links among crop diversification, microbial diversity, and soil organic carbon: Mini review and case studies

Rachel Wooliver, Stephanie N. Kivlin, Sindhu Jagadamma

***Methods S1****: Description of crop diversification case studies, microbial sequence processing and taxonomy assignment, and statistical analysis of soil microbial diversity and SOC*

1. Case study descriptions

In the main text of this manuscript, we provide results of five case studies that characterize soil microbial community and SOC responses to crop diversification (**Table 1** of the main text), and here we give a brief description of the studies. Ashworth et al. (2017) reported results of two no-till experiments that included crop rotation and cover cropping treatments at two locations in the southeastern United States and analyzed microbial community responses to these treatments in two sequential years. Cloutier et al. (2020) compared soil fungal communities among nine cover cropping treatments varying from one species to a six-species mixture, analyzing cover cropping effects within each of two consecutive seasons (spring and summer). Gao et al. (2019) implemented an agroforestry experiment, comparing soil bacterial communities in a conventional wheat monoculture to those of a chronosequence of walnut-wheat systems. Song et al. (2018) characterized both fungal and bacterial soil community responses to crop rotation, including two diversity controls: fallow soybean and continuous soybean. Last, Strom et al. (2020) is the study of longest experimental duration among the case studies (33-34 years), focusing on fungal community responses to crop rotation and in each of three seasons within two consecutive sampling years. We include only four crop system treatments in our analysis (out of the 16 from the original study) to isolate effects of crop diversity treatments that are most commonly used by producers. Specifically, we include only monoculture vs. annual rotation of Bt corn and soybean-cyst-susceptible soybean. We exclude plots sampled in each year of a five-year corn-soybean rotation, along with monoculture plots of non-Bt corn and non-soybean-cyst-susceptible soybean.

2. Sequence processing and taxonomy assignment

All analyses were implemented in R statistical software version 4.0.3 (R Core Team, 2020), and all R codes are available at (https://github.com/rwoolive/microbe-cropdiv). After downloading sequence data from NCBI or obtaining from the corresponding author, we deinterleaved sequence files when necessary using BBMap version 38.90 (<https://sourceforge.net/projects/bbmap/>). Then, we used the DADA2 pipeline version 1.16 (Callahan et al., 2016; <https://benjjneb.github.io/dada2/index.html>) in R to produce abundance tables of amplicon sequence variants (ASVs). Different ASVs represent gene sequences that differ by at least one nucleotide, compared to operational taxon units (OTUs) which represent gene sequences that share at least a set percentage of identical nucleotides (usually 97%). The advantage of the ASV approach over the OTU approach is that it is repeatable, however the approaches yield the same results for diversity and composition (Glassman & Martiny, 2018). Default parameters were used for sequence processing through the DADA2 pipeline, except we adjusted parameters for filtering and trimming according to each study’s read quality and primer lengths. After removing chimeras and ASVs with less than 10 reads, we assigned taxonomy using the RDP online tool with 95% confidence threshold (<http://rdp.cme.msu.edu/classifier/classifier.jsp>; Wang et al., 2007). We removed ASVs assigned to non-target taxa, which consisted of only *Zea mays* (38 in Ashworth et al., 2017; 4 in Song et al., 2018). ASV abundance tables were then transformed to proportional abundance within each sample. Though other normalization approaches are available, we used proportional abundance because it is repeatable and yields the most accurate results at low effect sizes (Weiss et al., 2017).

3. SOC and microbial community analysis

All analysis was conducted in R (R Core Team, 2020). We quantified soil microbial diversity for each sample within each dataset using the inverse Simpson diversity index. To determine how crop diversification alters SOC and microbial diversity relative to low-diversity controls, we calculated treatment effect sizes using log response ratios and 95% confidence intervals that include a correction factor for small sample sizes (Pustejovsky, 2018). If 95% confidence intervals did not include zero, diversification treatment effects were considered significant. We performed additional analysis on subsetted fungal datasets (according to Nguyen et al., 2016) to determine if fungal functional groups respond differently to crop diversification. If plot-level data on SOC were not available, we calculated an effect size and determined significance based on treatment means and standard deviations that were reported in the original study. For experimental designs that included temporal factors (year and/or season of soil sampling; Ashworth et al., 2017; Cloutier et al., 2020; Strom et al., 2020) or two-way diversification treatments (Ashworth et al., 2017), we used linear mixed effects models (implemented in the nlme package, version 3.1-153; Pinheiro et al. 2021) to test for all possible interactions (results are reported in **Table S2**). In these models, replicate was included as a random effect and, where necessary, split-plot factors were nested within replicate. If significant (alpha<0.05) interactions were detected, then separate effect sizes were calculated for subsetted data. For the Ashworth et al. (2017) dataset, the two locations (RECM and MTREC) were analyzed separately to explore site-specific responses. Effect sizes are shown in **Figs. 1** (bacterial diversity)**, 2** (SOC)**,** and **3** (fungal diversity) of the main text.

To determine crop diversification treatment effects on soil microbial community composition, we used permutational analysis of variance (PERMANOVA, implemented in the vegan package, version 2.5-7; Oksanen et al., 2020). Analyses were based on Bray-Curtis distances and included using field replicate as a random effect, ASV abundance table as the response variable, and crop diversification as the predictor variable. When the study design included two-way diversification treatments and/or temporal factors such as season or year of sampling, these were included as predictor variables with all possible interactions. Results are reported in **Table S3**.

To determine relationships between SOC and microbial communities, we used linear mixed models and distance-based redundancy analysis. We extracted standardized beta values and associated p-values from linear mixed models (nlme package, version 3.1-153; Pinheiro et al. 2021) that included field replicate as a random effect, scaled SOC as the response variable, and scaled inverse Simpson diversity as the predictor variable. Standardized betas are displayed in **Fig. 5** of the main text. Then, we implemented redundancy analysis based on Jaccard distance (vegan package, version 2.5-7; Oksanen et al., 2020), using field replicate as a random effect, ASV abundance table as the response variable, and SOC as the predictor variable. Readily available carbon (quantified as either dissolved organic carbon or permanganate-oxidizable carbon) was included as an additional predictor variable when available. Significance of SOC as a predictor of microbial communities was then determined using permutational analysis of variance. Redundancy analyses are displayed in **Figs. 4** (bacterial communities) and **6** (fungal communities) of the main text.

References

Ashworth, A. J., DeBruyn, J. M., Allen, F. L., Radosevich, M., and Owens, P. R. (2017). Microbial community structure is affected by cropping sequences and poultry litter under long-term no-tillage. Soil Biol. Biochem. 114, 210–219. doi:10.1016/j.soilbio.2017.07.019.

Callahan, B. J., McMurdie, P. J., Rosen, M. J., Han, A. W., Johnson, A. J. A., and Holmes, S. P. (2016). DADA2: High-resolution sample inference from Illumina amplicon data. Nat. Methods 13, 581–583. doi:10.1038/nmeth.3869.

Cloutier, M. L., Murrell, E., Barbercheck, M., Kaye, J., Finney, D., García-González, I., et al. (2020). Fungal community shifts in soils with varied cover crop treatments and edaphic properties. Sci. Rep. 10, 6198. doi:10.1038/s41598-020-63173-7.

Gao, P., Zheng, X., Wang, L., Liu, B., and Zhang, S. (2019). Changes in the soil bacterial community in a chronosequence of temperate walnut-based intercropping systems. Forests 10, 299. doi:10.3390/f10040299.

Glassman, S. I., and Martiny, J. B. H. (2018). Broadscale ecological patterns are robust to use of exact sequence variants versus operational taxonomic units. mSphere 3, e00148-18. doi:10.1128/mSphere.00148-18.

Nguyen, N. H., Song, Z., Bates, S. T., Branco, S., Tedersoo, L., Menke, J., et al. (2016). FUNGuild: An open annotation tool for parsing fungal community datasets by ecological guild. Fungal Ecol. 20, 241–248. doi:10.1016/j.funeco.2015.06.006.

Oksanen, J., Blanchet, F. G., Friendly, M., Kindt, R., Legendre, P., McGlinnm, D., Minchin, P. R., O'Hara, R. B., Simpson, G. L., Solymos, P., Stevens, M. H. H., Szoecs, E., Wagner, H. (2020). vegan: Community Ecology Package. R package version 2.5-7. <https://CRAN.R-project.org/package=vegan>.

Pinheiro, J., Bates, D., DebRoy, S., Sarkar, D., R Core Team (2021). nlme: Linear and Nonlinear Mixed Effects Models. R package version 3.1-153, <https://CRAN.R-project.org/package=nlme>.

Pustejovsky, J. E. (2018). Using response ratios for meta-analyzing single-case designs with behavioral outcomes. J. of Sch. Psych. 68, 99–112. doi:10.1016/j.jsp.2018.02.003.

R Core Team (2020). R: a language and environment for statistical computing. R Foundation for Statistical Computing, Vienna.

Song, X., Tao, B., Guo, J., Li, J., and Chen, G. (2018). Changes in the microbial community structure and soil chemical properties of vertisols under different cropping systems in northern China. Front. Environ. Sci. 6, 132. doi:10.3389/fenvs.2018.00132.

Strom, N., Hu, W., Haarith, D., Chen, S., and Bushley, K. (2020). Interactions between soil properties, fungal communities, the soybean cyst nematode, and crop yield under continuous corn and soybean monoculture. Appl. Soil Ecol. 147, 103388. doi:10.1016/j.apsoil.2019.103388.

Weiss, S., Xu, Z. Z., Peddada, S., Amir, A., Bittinger, K., Gonzalez, A., et al. (2017). Normalization and microbial differential abundance strategies depend upon data characteristics. Microbiome 5, 27. doi:10.1186/s40168-017-0237-y.

## **Table S1**: Sequence data across case studies. Listed are DNA primers, sequencing depth information (read length in base pairs and total number of reads after processing), total number of ASVs identified, and relative abundances by taxonomic/functional group (ordered from highest to lowest abundance).

|  | **Ashworth et al. 2017** | **Cloutier et al. 2020** | **Gao et al. 2019*** | **Song et al. 2018** | **Strom et al. 2020** |
| --- | --- | --- | --- | --- | --- |
| **Primers (gene region)** | **Bacteria**: 341F/785R (V3-V4) | **Fungi**: ITS1F/ITS2R (ITS) | **Bacteria:** 319F/806R (V3-V4) | **Bacteria**: 515F/907R (V4-V5)  **Fungi**: ITS1F/ITS1R (ITS1) | **Fungi**: ITS1F/ITS1R (ITS1) |
| **Paired-end Illumina read length** | 250 bases | 250 bases | 300 bases | 250 bases | 250 bases |
| **# reads after processing** | **Bacteria**  Total: 14,901,055  Per sample: 155,219 | **Fungi**  Total: 1,480,374  Per sample: 20,561 | **Bacteria**  Total: 173,675  Per sample: 14,473 | **Bacteria**  Total: 2,339,233  Per sample: 194,936  **Fungi**  Total: 2,894,805  Per sample: 241,234 | **Fungi**  Total: 7,697,025  Per sample: 80,177 |
| **Average Good’s coverage** | **Bacteria**: 99.97% | **Fungi**: 92.24% | **Bacteria**: 100% | **Bacteria**: 99.97%  **Fungi**: 100% | **Fungi**: 99.98% |
| **Total # OTUs from original paper** | **Bacteria:** Not stated | **Fungi:** 12,275 | **Bacteria:** 6,504 | **Bacteria:** 6,879  **Fungi**: 3,038 | **Fungi:** 8,587 |
| **Total # ASVs** | **Bacteria**: 77,507 (+66 arch.) | **Fungi**: 14,701 | **Bacteria:** 2,921 (+7 arch.) | **Bacteria**: 17,674 (+57 arch.)  **Fungi**: 4,557 | **Fungi**: 9,290 |
| **Fungal taxonomic groups** (average relative abundance) | *NA* | Ascomycota (32.3%)  Basidiomycota (32.2%)  Zygomycota (22.1%)  Chytridiomycota (0.7%)  Glomeromycota (0.3%) | *NA* | Ascomycota (66.5%)  Basidiomycota (16.9%)  Zygomycota (11.5%)  Chytridiomycota (1.5%)  Glomeromycota (0.4%) | Ascomycota (61.7%)  Basidiomycota (14.2%)  Zygomycota (13.2%)  Chytridiomycota (3.8%)  Glomeromycota (1.6%) |
| **Fungal functional guilds** (average relative abundance) | *NA* | Saprotroph (46.6%)  Plant pathogen (10.8%)  AMF (0.3%) | *NA* | Saprotroph (41.2%)  Plant pathogen (22.6%)  AMF (0.2%) | Saprotroph (39.2%)  Plant pathogen (9.8%)  AMF (0.8%) |
| **Bacterial taxonomic groups** (average relative abundance) | Acidobacteria (22.4%)  Proteobacteria (22%)  *α-proteobacteria (9.2%)*  *β-proteobacteria (5.9%)*  *γ-proteobacteria (4.2%)*  Actinobacteria (15.4%)  Firmicutes (7.6%)  Planctomycetes (5.5%)  Chloroflexi (5.2%)  Verrucomicrobia (5%)  Bacteroidetes (3.7%)  Gemmatimonadetes (3%) | *NA* | Firmicutes (38.5%)  Proteobacteria (21.9%)  *α-proteobacteria (11.2%)*  *β-proteobacteria (4.8%)*  *γ-proteobacteria (2.7%)*  Acidobacteria (14.4%)  Actinobacteria (14.3%)  Gemmatimonadetes (5.2%)  Bacteroidetes (1.3%)  Chloroflexi (1.3%)  Verrucomicrobia (0.5%)  Planctomycetes (0.1%) | Proteobacteria (28.1%)  *β-proteobacteria (9.9%)*  *α-proteobacteria (7.9%)*  *γ-proteobacteria (7.4%)*  Acidobacteria (22%)  Bacteroidetes (19%)  Actinobacteria (9.8%)  Chloroflexi (3.6%)  Gemmatimonadetes (3.3%)  Planctomycetes (2.9%)  Verrucomicrobia (1.8%)  Firmicutes (1.3%) | *NA* |

*** Reads were poor quality, dropping below quality scores of 30 at 200 and 90 respectively for forward and reverse reads**

##

## **Table S2**: Effects of crop diversification on soil microbial Inverse Simpson diversity and soil carbon across case studies. Shown are results of ANOVA. Significant effects at α=0.05 are bolded.

| **Study** | **Bacterial diversity** | **Fungal diversity** | **Soil carbon** |
| --- | --- | --- | --- |
| **Ashworth MTREC** | Cropsys: Chisq_Df_=2.914_2_, p=0.233  **Cover: Chisq_Df_=8.595_2_, p=0.014**  Year: Chisq_Df_=2.49_1_, p=0.115  Cropsys*Cover: Chisq_Df_=2.89_4_, p=0.576  Cropsys*Year: Chisq_Df_=0.005_2_, p=0.997  Cover*Year: Chisq_Df_=0.173_2_, p=0.917  Cropsys*Cover*Year: Chisq_Df_=0.94_4_, p=0.919 |  | Cropsys: Chisq_Df_=4.546_2_, p=0.103  **Cover: Chisq_Df_=9.056**_2_**, p=0.011**  Year: Chisq_Df_=0.009_1_, p=0.923  **Cropsys*Cover: Chisq_Df_=13.09_4_, p=0.011 ^A^**  Cropsys*Year: Chisq_Df_=0.237_2_, p=0.888  Cover*Year: Chisq_Df_=0.113_2_, p=0.945  Cropsys*Cover*Year: Chisq_Df_=0.264_4_, p=0.992 |
| **Ashworth RECM** | Cropsys: Chisq_Df_=2.838_2_, p=0.242  Cover: Chisq_Df_=5.529_2_, p=0.063  **Year: Chisq_Df_=14.894_1_, p<0.01**  Cropsys*Cover: Chisq_Df_=2.878_4_, p=0.578  Cropsys*Year: Chisq_Df_=2.436_2_, p=0.296  **Cover*Year: Chisq_Df_=6.306_2_, p=0.043**  Cropsys*Cover*Year: Chisq_Df_=1.621_4_, p=0.805 | *NA* | **Cropsys: Chisq_Df_=6.439_2_, p=0.04**  Cover: Chisq_Df_=1.552_2_, p=0.46  Year: Chisq_Df_=0_1_, p=1  Cropsys*Cover: Chisq_Df_=3.658_4_, p=0.454  Cropsys*Year: Chisq_Df_=0_2_, p=1  Cover*Year: Chisq_Df_=0_2_, p=1  Cropsys*Cover*Year: Chisq_Df_=0_4_, p=1 |
| **Cloutier** | *NA* | Cover: Chisq_Df_=11.216_8_, p=0.19  **Season: Chisq_Df_=11.035_1_, p=0.001**  Cover*Season: Chisq_Df_=10.188_8_, p=0.252 | **Cover: Chisq_Df_=20.159_8_, p=0.01**  Season: Chisq_Df_=0_1_, p=1  Cover*Season: Chisq_Df_=0_8_, p=1 |
| **Gao** | **Cropsys: Chisq_Df_=9.235_3_, p=0.026** | *NA* | *NA (Only treatment-level mean data provided)* |
| **Song** | Cropsys: Chisq_Df_=7.64_3_, p=0.054 | **Cropsys: Chisq_Df_=11.067_3_, p=0.011** | *NA (Only treatment-level mean data provided)* |
| **Strom** | *NA* | **Cropsys: Chisq_Df_=9.44_3_, p=0.024**  Year: Chisq_Df_=3.575_1_, p=0.059  Season: Chisq_Df_=3.991_2_, p=0.136  Cropsys*Year: Chisq_Df_=2.226_3_, p=0.527  Cropsys*Season: Chisq_Df_=11.171_6_, p=0.083  Year*Season: Chisq_Df_=2.411_2_, p=0.299  Cropsys*Year*Season: Chisq_Df_=5.032_6_, p=0.54 | **Cropsys: Chisq_Df_=57.038_3_, p<0.01**  Year: Chisq_Df_=3.796_1_, p=0.051  Season: Chisq_Df_=0_2_, p=1  **Cropsys*Year: Chisq_Df_=11.607_3_, p=0.009**  Cropsys*Season: Chisq_Df_=0_6_, p=1  Year*Season: Chisq_Df_=0_2_, p=1  Cropsys*Year*Season: Chisq_Df_=0_6_, p=1 |

^A^ This interaction is driven by a difference between cover treatments (vetch vs. wheat) under corn monoculture, rather than between either cover treatment and control fallow.

## **Table S3:** Effects of crop diversification on soil microbial community composition across case studies. Shown are results of PERMANOVA. Significant effects at α=0.05 are bolded.

| **Study** | **Bacteria** | **Fungi** |
| --- | --- | --- |
| **Ashworth MTREC** | **Cropsys: F_Df_=1.85_3_, R^2^=0.05, p<0.01**  **Cover: F_Df_=1.67_3_, R^2^=0.05, p<0.01**  **Year: F_Df_=7.94_1_, R^2^=0.07, p<0.01**  Cropsys*Cover: F_Df_=1.07_9_, R^2^=0.09, p=0.14  Cropsys*Year: F_Df_=1.07_3_, R^2^=0.03, p=0.23  Cover*Year: F_Df_=0.91_3_, R^2^=0.03, p=0.69  Cropsys*Cover*Year: F_Df_=0.93_9_, R^2^=0.08, p=0.74 |  |
| **Ashworth RECM** | **Cropsys: F_Df_=1.85_3_, R^2^=0.05, p<0.01**  **Cover: F_Df_=1.67_3_, R^2^=0.05, p<0.01**  **Year: F_Df_=7.94_1_, R^2^=0.07, p<0.01**  Cropsys*Cover: F_Df_=1.07_9_, R^2^=0.09, p=0.14  Cropsys*Year: F_Df_=1.07_3_, R^2^=0.03, p=0.23  Cover*Year: F_Df_=0.91_3_, R^2^0.03, p=0.69  Cropsys*Cover*Year: F_Df_=0.93_9_, R^2^=0.08, p=0.74 | *NA* |
| **Cloutier** | *NA* | **Cover: F_Df_=1.23_8_, R^2^=0.13, p<0.01**  **Season: F_Df_=2.92_1_, R^2^=0.04, p<0.01**  Cover*Season: F_Df_=0.95_8_, R^2^=0.1, p=0.53 |
| **Gao** | **Cropsys: F_Df_=1.98_3_, R^2^=0.43, p=0.02** | *NA* |
| **Song** | **Cropsys: F_Df_=6.16_3_, R^2^=0.7, p<0.01** | **Cropsys: F_Df_=7.32_3_, R^2^=0.73, p<0.01** |
| **Strom** | *NA* | **Cropsys: F_Df_=1.65_3_, R^2^=0.05, p<0.01** |
